# Tenofovir-Containing Antiretroviral Therapy and Clinical Outcomes of SARS-CoV-2 Infection in People Living with HIV

**DOI:** 10.3390/v15051127

**Published:** 2023-05-09

**Authors:** María F. Rombini, Diego Cecchini, Sofía Diana Menendez, Liliana Calanni, Rosana Cuini, Elena Obieta, María M. Greco, Fabricio Morales, Laura Morganti, Claudia Migazzi, Yasmin El Kozah, Pablo Parenti, Isabel Cassetti, on behalf of the COVIDARE Study Team

**Affiliations:** 1 Helios Salud, Buenos Aires 1141, Argentina; 2 Hospital Cosme Argerich, Buenos Aires 1155, Argentina; 3 CEIN Unidad Infectológica Neuquén, Neuquén 8300, Argentina; 4 Hospital Teodoro Álvarez, Buenos Aires 1406, Argentina; 5 Hospital Municipal Ciudad de Boulogne, Boulogne 1609, Argentina; 6 Hospital Español de La Plata, La Plata 1902, Argentina; 7 IPTEI, Buenos Aires 1415, Argentina; 8 Hospital Presidente Perón de Avellaneda, Avellaneda 1872, Argentina; 9 CAICI, Rosario 2000, Argentina

**Keywords:** COVID-19, HIV, tenofovir, hospitalization, oxygen, antiretroviral therapy

## Abstract

Tenofovir has been hypothesized to be effective against COVID-19 and is available as two prodrugs, tenofovir disoproxil fumarate (TDF) and tenofovir alafenamide (TAF), both part of antiretroviral therapy (ART) regimens. People living with human immunodeficiency virus (PLWH) might be at higher risk for COVID-19 progression; however, information about the impact of tenofovir on COVID-19 clinical outcomes remains controversial. The COVIDARE is a prospective observational multicentric study in Argentina. PLWH with COVID-19 were enrolled from September 2020 to mid-June 2022. Patients were stratified according to baseline ART into those with tenofovir (TDF or TAF) and those without. Univariate and multivariate analyses were performed to evaluate the impact of tenofovir vs. non-tenofovir-containing regimens on major clinical outcomes. Of the 1155 subjects evaluated, 927 (80%) received tenofovir-based ART (79% TDF, 21% TAF) whilst the remaining population was under non-tenofovir regimens. The non-tenofovir group had older age and a higher prevalence of heart and kidney disease. Regarding the prevalence of symptomatic COVID-19, tomographic findings, hospitalization, and mortality, no differences were observed. The oxygen therapy requirement was higher in the non-tenofovir group. In the multivariate analyses, a first model with adjustment for viral load, CD4 T-cell count, and overall comorbidities showed that oxygen requirement was associated with non-tenofovir ART. In a second model with adjustment by chronic kidney disease, tenofovir exposure was not statistically significant.

## 1. Introduction

Since the beginning of the COVID-19 pandemic, severe acute respiratory syndrome coronavirus 2 (SARS-CoV-2) has infected more than 600 million people, leading to more than 6.5 million deaths around the world. In Argentina, the number of infected people as of February 2023 was 10 million, and more than 130,000 related deaths were reported [1]. Despite this being an unprecedented global public health challenge, therapeutic options are paradoxically limited, even in critical cases.

According to the WHO living guideline on therapeutics for COVID-19, a few immune modulators and antiviral agents are recommended for severe cases. These include a strong recommendation for the use of systemic corticosteroids, interleukin-6 (IL-6) receptor blockers (tocilizumab or sarilumab), the Janus kinase (JAK) inhibitor baricitinib, and a conditional recommendation for remdesivir. In patients with non-severe COVID-19 at the highest risk of hospitalization, a strong recommendation for nirmatrelvir–ritonavir and a conditional recommendation for molnupiravir and remdesivir is provided. Other agents that were initially considered therapeutic options, such as ivermectin and colchicine, are currently not recommended [2].

The potential role of antiretroviral drugs as a protective factor to prevent SARS-CoV-2 infection or progression to severe disease has been controversial. Early in the pandemic, the protease inhibitor lopinavir/ritonavir was empirically indicated in hospitalized COVID-19 patients, but its use had to be discontinued due to a lack of evidence of effectiveness in clinical trials [3]. The nucleotide analog tenofovir has been hypothesized to be potentially effective against SARS-CoV-2 [4,5,6]. Tenofovir is available as two prodrugs, tenofovir disoproxil fumarate (TDF) and tenofovir alafenamide (TAF), both of them currently an essential part of antiretroviral therapy (ART) regimens as so-called backbones. In vitro studies have shown that tenofovir inhibits RNA-dependent RNA polymerase, an indispensable enzyme for SARS-CoV-2 replication, but its clinical efficacy in patients with COVID-19 remains uncertain [7]. Evidence supporting tenofovir-based strategies either for treatment or prevention of COVID-19 remains sparse, and thus a controversial issue in the literature. Different cohorts reported that people treated with TDF for HIV were less likely to develop SARS-CoV-2 infection and/or severe COVID-19 [8,9,10]. Moreover, regarding patients with hepatitis B and COVID-19, better outcomes were described in those on therapy with TDF vs. entecavir [11].

People living with human immunodeficiency virus (PLWH) might be at particularly high risk for severe COVID-19 progression [12,13,14]. HIV infection is among the chronic conditions to be considered in COVID-19 vaccine prioritization, as per WHO criteria [15,16,17]. According to official statistics, Argentina has about 136,000 HIV-infected patients and a long history of free ART delivery since 1992, and both TDF and TAF are available as part of combined ART, for either health-insured or non-insured patients [18].

In this context, we aim to describe associated clinical outcomes and disease severity of COVID-19 in PLWH from a national cohort in Argentina that is receiving tenofovir and non-tenofovir as the so-called backbone of their ongoing ART regimen.

## 2. Materials and Methods

### 2.1. Study Design

The COVIDARE (COVID-19 Argentine Registry) study is a multicenter prospective cohort study carried out in public and private hospitals and HIV outpatient clinics in Argentina, designed to assess the clinical and epidemiological characteristics of PLWH coinfected with SARS-CoV-2 within the country. Centers were located in the Buenos Aires Metropolitan Area, Neuquén, and Rosario. Institutional review board approval in each participating site was obtained before activation for this study.

### 2.2. Participants

Study staff in each participating center enrolled HIV-infected patients with COVID-19 diagnosis on routine care basis either through in-person visits (both for outpatients and/or during hospitalization) or through mobile devices as a video consultation (in institutions with telemedicine services). Informed consent was obtained for each patient before enrollment. Given the non-interventional nature of the study, the institutional review board of certain participating sites waived the request for written informed consent, with both oral and written consent being acceptable for conducting this research in the pandemic lockout context.

PLWH ≥ 18 years assisted at any of the participating centers with confirmed SARS-CoV-2 infection were eligible. HIV infection status was confirmed based on the previous history of positive ELISA plus Western blot or positive ELISA plus a detectable viral load, according to local and international guidelines. Reverse transcriptase polymerase chain reaction (PCR), antigen rapid test (Ag), or any other method validated by the Argentine Ministry of Health at the time of enrollment was accepted to confirm SARS-CoV-2 infection. Diagnosis based on SARS-CoV-2 serology (IgG or IgM) by ELISA or immunochromatography or epidemiologic criteria according to Ministry of Health guidelines was also acceptable as proof of COVID-19 infection [19].

For this analysis, PLWH with ongoing ART were eligible for inclusion if they had been enrolled by any of the participating sites between September 2020 and June 2022. The study allowed patients to be included retrospectively after the episode of COVID-19; the first documented case of the cohort was from 1 March 2020, and the last was from 9 June 2022. Patients were excluded if complete information on hospitalization admission and key variables of interest were not available or if they were not on ART at the time of COVID-19 diagnosis.

### 2.3. Data Collection

Data were collected from the REDCap database (Research Electronic Data Capture, Vanderbilt University, Nashville, TN, USA) hosted by Fundación Helios Salud.

Data capture included demographic profile (age, sex at birth), epidemiologic variables regarding HIV infection, immune and virological profile (viral load, CD4 T-cell count), ART, additional comorbidities (chronic kidney disease, heart disease, obesity, hypertension, and diabetes, among others), tomographic findings, COVID-episode outcomes (hospitalization, oxygen requirement, COVID-19 therapy prescription, mortality), and COVID-19 immunization status (at least one dose of any COVID-19 approved vaccine in Argentina).

### 2.4. Statistical Analyses

The participants were grouped according to baseline tenofovir- or non-tenofovir-containing ART, considering demographic characteristics, symptomatic disease, tomographic abnormalities, history of COVID-19 vaccination, and the following major clinical outcomes: hospitalization, oxygen supplementation, COVID-19 therapy requirement, and mortality. Categorical variables were described using absolute and relative frequencies and compared by the X^2^ test regarding group differences. Continuous variables were described using medians with interquartile ranges (IQRs) and compared by the Mann–Whitney U test for differences between groups. All tests were two-sided, and a *p*-value less than 0.05 was considered significant. Variables with missing values were excluded from the analysis.

Simple logistic regression was utilized to evaluate the association between the different variables of interest (history of vaccination with COVID-19, CD4 T-cell count <350 cells/μL, age, and comorbidities) and the main clinical outcomes with differences between the groups. The odds ratio (OR) with a 95% confidence interval (CI) was used as a measure of association.

Then, two multivariable multiple logistic regression analysis models were used to adjust the results for possible confounding factors. In the first model, associated comorbidities were globally analyzed. In a second model, those comorbidities that were not balanced between groups (heart failure, chronic renal failure, and chronic corticosteroid use) were introduced separately. Age was introduced into both models as a continuous variable.

The concordance and discrimination of both models were tested with the Hosmer–Lemeshow test and ROC curve analysis. STATA/MP 14.0 for Windows was used for data analysis. Furthermore, separate comparisons were made between cohorts with TDF and cohorts without TDF (TAF + other ART groupings).

## 3. Results

**Figure 1:**
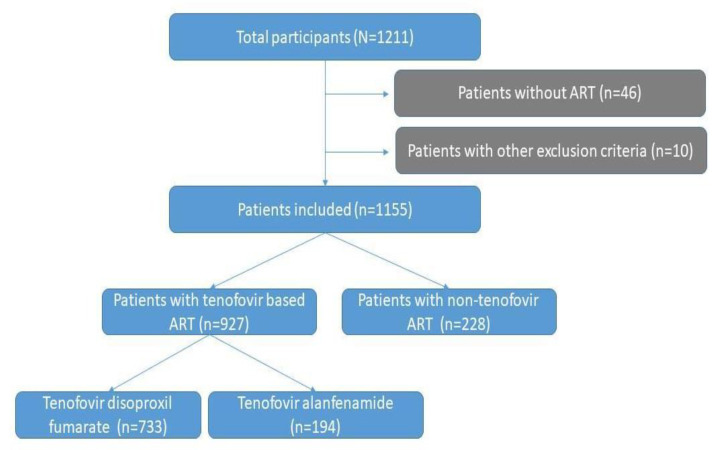
Flowchart of HIV/COVID-19-infected patients according to antiretroviral therapy (ART) status in the COVIDARE cohort, Argentina (2020–2022).

Of the 1211 eligible participants, 1155 patients met inclusion and had no exclusion criteria for primary analysis, as shown in Figure 1. Of them, 927 (80%) had ongoing tenofovir-based ART (79% TDF, 21% TAF). Conversely, 228 were under non-tenofovir ART.

Accompanying drugs included almost universally XTC (either 3TC or FTC) and, as a third drug, mainly bictegravir (77%) for TAF regimens, and darunavir/ritonavir (DRV/r, 39.6%), efavirenz (EFV, 32%), or dolutegravir (DTG, 14.4%) for those with TDF. Non-tenofovir ART was predominantly based on abacavir (ABC) + 3TC (63.5%) or single 3TC (19%; mostly dual therapy regimens) as a nucleoside analog, plus DTG (32%), DRV/r (34.6%), or EFV (20%), among others.

SARS-CoV-2 infection diagnosis was based on studies of respiratory secretions by PCR (82.9%) or Ag (11.2%). In a smaller proportion of cases, the diagnosis was based on epidemiological criteria (2%) and serology (2.6%).

### 3.1. Tenofovir vs. Non-Tenofovir Group Comparison

The demographic characteristics, comorbidities, co-medications, immune and virological status, prevalence of tomographic abnormalities, hospitalization, oxygen supplementation requirement, vaccination status, and mortality are described in Table 1.

Considering demographics, both groups were similar except for older age in the non-tenofovir group. The non-tenofovir group had a higher CD4 count. The overall prevalence of comorbidities was similar between groups, but a higher prevalence of chronic kidney disease, chronic steroid use, and cardiac insufficiency was observed in the non-tenofovir cohort. The prevalence of symptomatic disease and COVID-19 therapy requirements were similar between the tenofovir and non-tenofovir groups. COVID-19 therapy included steroids (78.8%), azithromycin (37.9%), lopinavir/ritonavir (2.3%), convalescent plasma (9.1%), hydroxychloroquine (1.5%), colchicine (0.8%), and ivermectin (0.8%), without differences between groups.

Regarding overall tomographic abnormalities, no differences were observed between cohorts. The most prevalent findings were ground-glass opacities (79.3%), followed by consolidation patterns (13.4%) and pleural effusion (2.4%).

Vaccination (at least one dose of any SARS-CoV-2 vaccine) was higher in the non-tenofovir group. Oxygen therapy was required in 9.9% of patients (n = 114), with a higher prevalence in the non-tenofovir group (*p* 0.03), even when vaccinated patients were excluded (data not shown). Overall hospitalization and mortality were 16% (n = 185) and 1.1% (n = 13), respectively, similar between tenofovir and non-tenofovir cohorts.

### 3.2. Model 1: Variables Associated with the Requirement of Oxygen Supplementation

Univariate analysis was performed to identify significant associations between oxygen requirement and different potential risk variables. The comorbidities were included globally (Table 2).

The requirement of oxygen therapy was associated with older age, the presence of comorbidities, low CD4 count (<350 cells/μL), and non-tenofovir therapy. In contrast, it was inversely associated with a history of vaccination for COVID-19 with at least one dose. In multivariable analysis, immunization history, age, and a low CD4 count were confounders of the association between non-tenofovir therapy and supplemental oxygen requirement. Treatment without tenofovir adjusted for the other variables was significantly associated with an increased risk of oxygen requirement (OR 2.2; *p* 0.014), as shown in Table 3. The model demonstrated adequate calibration and discrimination.

### 3.3. Model 2: Evaluation of Comorbidities Associated with the Requirement of Oxygen Supplementation

Univariate analysis was performed including the comorbidities with the highest prevalence in the non-tenofovir group, namely chronic kidney disease, chronic steroid use, and heart failure, separately to assess their association with oxygen requirement (Table 4).

The requirement of oxygen supplementation was associated with the presence of chronic kidney disease, heart failure, and a group of other comorbidities.

In the multivariable analysis of this model, chronic renal failure was shown to be a confounding factor in the association between non-tenofovir treatment and oxygen requirement. When the presence of chronic renal failure was entered into the model, treatment was not statistically significant.

In this adjusted multivariable analysis, older age, the presence of chronic renal failure, other comorbidities, and low CD4 count (<350 cells/μL) were significant risk factors and immunization history was a protective factor, as shown in Table 5. Non-tenofovir ART was not associated with supplemental oxygen requirement. The model demonstrated adequate calibration and discrimination.

### 3.4. TDF vs. Non-TDF Group Comparison

**Figure A1:**
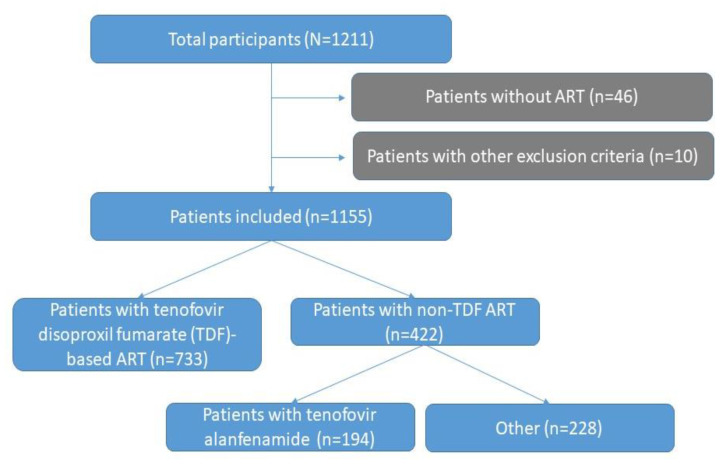
Flowchart of HIV/COVID-19-infected patients according to antiretroviral therapy (ART) status in the COVIDARE cohort, Argentina (2020–2022).

An additional comparison between TDF (n = 733) and non-TDF (including TAF; n = 422) groups was performed (Appendix A, Figure A1). The non-TDF group was significantly older and had a higher immunization rate. No statistically significant differences were observed in terms of hospitalization, oxygen supplementation, COVID-19 therapy requirement, and mortality (Appendix A, Table A1).

## 4. Discussion

Our study is the first Latin American cohort to analyze the impact of baseline ART in clinical outcomes of PLWH with SARS-CoV-2 infection, with a focus on tenofovir as part of nucleos(t)ide inhibitor (NRTI) backbone therapy. Despite model 1, “overall comorbidities”, showing that a non-tenofovir ART is a risk factor for oxygen requirement in PLWH with COVID-19, when chronic kidney disease was specifically included (in model 2), treatment was not statistically significant.

While several studies have established HIV infection itself as a risk factor for poor COVID-19-related outcomes in terms of hospitalization and mortality, mainly associated with advanced stage of immunosuppression and comorbidities [12,13,14,20,21,22], others have not found this association [23,24,25,26]. Although protease inhibitors such as lopinavir/ritonavir and darunavir had been initially considered as potential candidates for COVID-19 therapy, studies showed a lack of efficacy for the prevention of infection or worsening of respiratory symptoms [27,28]. Considering NRTIs, tenofovir is active as an inhibitor of SARS-CoV-2 RNA-dependent RNA polymerase [7,29]. However, its clinical role in the prevention and potential treatment of COVID-19 remains controversial, including any differences between the “old” TDF and TAF. In a cohort study in Spain describing the incidence and severity of COVID-19 in HIV-positive persons receiving ART, Del Amo J. et al. found an approximately 50% lower risk of COVID-19 hospitalization among those using TDF/FTC compared to those on TAF/FTC and other NRTI combinations, but the analysis did not adjust for comorbidities [9,30]. In South Africa, a study found a nearly 60% lower risk of COVID-19 mortality with TDF/FTC vs. ABC or zidovudine after adjusting for kidney disease, viral suppression, and ART duration [10]. Similarly, a study among individuals with hepatitis B in Spain found that those on TDF had a lower risk of severe COVID-19 than those on entecavir [11]. In a recent publication, Li et al. compared the adjusted risks of documented SARS-CoV-2 infection, COVID-19-related hospitalization, and intensive care unit admission by baseline ART regimen in a cohort of men living with HIV. Compared with TAF/FTC, the estimated 18-month risk ratio of documented SARS-CoV-2 infection was 0.65 for TDF/FTC, 1.00 for ABC/3TC, and 0.87 for others. The corresponding risk ratios for COVID-19 hospitalization were 0.43, 1.09, and 1.21, suggesting a protective effect of TDF, but not TAF [8]. Conversely, Nomah et al. conducted a propensity score-matched analysis in the prospective PISCIS cohort of PLWH in Catalonia, Spain, and used adjusted Cox regression models to assess the association between tenofovir and SARS-CoV-2 outcomes. The authors concluded that neither TAF/FTC nor TDF/FTC were associated with reduced SARS-CoV-2 diagnosis rates or hospitalizations among PLWH [31]. In addition, a study on PrEP users demonstrated no difference in terms of clinical manifestations between people who received any tenofovir (TAF or TDF) and those who did not [32].

Considering such divisive results, we aimed to evaluate clinical outcomes according to tenofovir exposure status in PLWH in the COVIDARE study in Argentina, addressing an issue not previously studied in Latin America. Analysis was undertaken considering any tenofovir-containing ART vs. other regimens, and, in addition, considering TDF vs. non-TDF (including TAF) exposure. According to our results, hospitalization and mortality were similar between the tenofovir and non-tenofovir cohorts. However, oxygen therapy was required in 9.9% of patients, with a higher prevalence in the non-tenofovir group. Older age, overall comorbidities, low CD4 T-cell count, and non-tenofovir ART were associated with higher rates of oxygen requirement in the first model, but further adjustment mainly by chronic kidney disease showed no impact of tenofovir therapy in this outcome (model 2). These results add support to the literature suggesting no protective effect of tenofovir against severe SARS-CoV-2 infection and highlight the need of adjustment by specific comorbidities that are more prevalent in those PLWH without tenofovir exposure.

Oxygen requirement is an indisputable objective marker of disease severity less explored in the literature in comparison to hospitalization and mortality [8,23,24,25,30,33,34,35]. Our study registered cases documented since the beginning of the pandemic in Argentina, as early as March 2020. In the first months, some patients (particularly those more immunosuppressed) may have been hospitalized due to the HIV status itself, additional comorbidities, or isolation for epidemiological reasons more than the severity of COVID-19. Later on, when the local healthcare system approached its full capacity due to an evolving epidemic, some patients may have had oxygen supplementation on a non-hospitalized basis such as home care. Therefore, hospitalization may be a weaker marker of disease severity than oxygen requirement in our setting. On the other hand, mortality in our cohort was low, which may have prevented differences from being found between any groups.

Despite TDF and TAF both interfering with SARS-CoV-2 replication, TDF results in higher plasmatic tenofovir concentrations and bioavailability in tissues affected by COVID-19 [8]. We also explored if separating TDF from TAF would enhance or make visible any difference in clinical outcomes. When splitting TDF from TAF and including TAF with non-TDF regimens, no disparities were described for any outcome, suggesting no differences between them. In our cohort, the number of patients under TAF is relatively modest, as a reflection of real-life ART prescriptions in our country. In addition to a lack of impact of any tenofovir exposure on COVID-19 clinical outcomes, our results support that pharmacokinetic differences between TDF and TAF may not be relevant for these results.

Our study shows a real impact of certain comorbidities (mainly chronic kidney disease) on COVID-19 outcomes. As shown in Table 1, although no differences were described in the frequency of obesity, asthma, diabetes, and hypertension, comorbidities such as renal disease, heart failure, and chronic steroid use were more prevalent in the non-tenofovir group. This is attributable to the widely described potential detrimental effects of tenofovir disoproxil fumarate on renal function and bone mineral density [35], reflecting real-world clinical practice. Of note, removing TFA from the tenofovir-based group balanced the two groups in terms of renal disease issues (and other comorbidities), showing a lack of impact of therapy on major outcomes, as shown in Appendix A.

In addition, the patients in the non-tenofovir group were older and had a higher prevalence of vaccination against COVID-19. A higher prevalence of vaccination within this group is not surprising as older patients with comorbidities were a priority for the vaccination campaign in Argentina [36]. In both models, our study describes the protective effect of receiving at least one dose of any vaccine against SARS-CoV-2 in the requirement of oxygen supplementation. This finding supports the fundamental role of active immunization in preventing COVID-19’s worse clinical outcomes in this population. Conversely, a low CD4-T cell count (<350 cells/μL) remained in both models as a risk factor for oxygen supplementation, supporting the importance of adequate immunological control in these patients. This achievement depends mostly on adequate access and adherence to antiretroviral therapy, which leads to immune reconstitution.

Our study has several limitations that must be addressed. First, it was originally designed to describe clinical characteristics and outcomes of PLWH with COVID-19 without focusing on studying any impact of different ART drugs on this disease. Second, its observational nature may have prevented an adequate evaluation of additional undisclosed potential confounders. Third, it was undertaken exclusively in PLWH without a control group of the general population or other non-HIV-infected tenofovir-exposed groups, such as PrEP users, penalizing any comparison of the incidence of COVID-19. Despite these major limitations, our study is, to the best of our knowledge, the first one exploring the impact of any antiretroviral drug on COVID-19 outcomes in Latin America.

Despite ongoing controversy, tenofovir has not been studied in randomized clinical trials or, at least, in better-designed cohorts. Caution must be taken when analyzing outcomes as patients not exposed to tenofovir have a higher prevalence of comorbidities. Adjustment by chronic kidney disease must be particularly addressed because this population is particularly prone to worse clinical outcomes. Considering the ongoing SARS-CoV-2 pandemic, the emergence of new variants [37], and limited access to vaccination in several resource-constrained regions of the world [38], the study of antiviral molecules as either therapeutic or preventive agents should be prioritized to increase the current armamentarium against COVID-19.

## Figures and Tables

**Table 1:** Demographic profile, clinical characteristics, and outcomes of COVID-19 in HIV-infected patients with tenofovir- and non-tenofovir-based baseline therapy in Argentina (COVIDARE cohort, 2020–2022). Values are numbers (percentages) unless otherwise stated.

	TDF/TAF (n = 927)	Non-TDF/TAF (n = 228)	*p*
	n (%)	n (%)	
Male sex	612 (66)	145 (63.8)	0.54
Age, years (median; IQR)	44 (36–51)	50 (39–58)	<0.001
Comorbidities	349 (37.8)	93 (41.1)	0.35
Hypertension	107 (11.5)	36 (15.8)	0.08
Diabetes	54 (5.8)	20 (8.8)	0.10
Heart failure	3 (0.3)	5 (2.2)	0.002
Obesity	29 (12.7)	152 (16.4)	0.17
Asthma	17 (1.8)	3 (1.3)	0.59
Smoking habit	72 (7.8)	18 (7.9)	0.95
Chronic steroid use	1 (0.1)	4 (1.8)	0.001
Chronic kidney disease	5 (0.5)	16 (7)	<0.001
Virologic suppression (<20 copies/mL)	749 (81.1)	183 (80.3)	0.78
CD4 T-cell count, cells/μL (median; IQR)	597 (434–800)	656.5 (472.5–824)	0.04
Low CD4 count (<350 cells/μL)	147 (16)	22 (9.7)	0.02
COVID-19 vaccination (at least one dose)	193 (26.5)	54 (35.8)	0.02
Symptomatic COVID-19	888 (95.8)	221 (96.9)	0.42
Tomographic abnormalities (n = 139/1104) *	112 (12.7)	27 (12.3)	0.88
Hospitalization (n = 185/1106) *	144 (16.2)	41 (18.7)	0.37
Oxygen therapy (n = 114/1149) *	83 (9)	31 (13.8)	0.03
COVID-19 therapy (n = 132/1150) *	102 (11)	30 (13.3)	0.33
Mortality (n = 13/1148) *	8 (0.9)	5 (2.2)	0.09

* patients with event/patients with available data; TAF: tenofovir alenfenamide; TDF: tenofovir disoproxil fumarate; IQR: interquartile range.

**Table 2:** Variables associated with supplemental oxygen requirement (univariate analysis) in HIV-infected patients with COVID-19 in Argentina (COVIDARE cohort, 2020–2022). Comorbidities were considered globally.

Variable	OR (95% CI)	*p*
Non-tenofovir ART	1.63 (1.05–2.53)	0.03
Age (years)	1.05 (1.03–1.07)	<0.001
CD4 T-cell count (<350 cells/μL)	2.58 (1.65–4.04)	<0.001
Comorbidities	2.53 (1.67–3.85)	<0.001
History of COVID vaccination (at least one dose)	0.24 (0.1–0.55)	0.001

ART: antiretroviral therapy; OR: odds ratio.

**Table 3:** Model 1: multivariate analysis of variables associated with supplemental oxygen requirement in HIV-infected patients with COVID-19 in Argentina (COVIDARE cohort, 2020–2022). Comorbidities were considered globally.

Variable	OR	95% CI	*p*
Non-tenofovir ART	2.2	1.17–4.11	0.014
History of COVID-19 vaccination (at least one dose)	0.19	0.08–0.47	<0.001
CD4 T-cell count (<350 cells/μL)	3.97	2.20–7.16	<0.001
Age	1.04	1.01–1.06	0.008
Comorbidities	2.07	1.20–3.58	0.009

ART: antiretroviral therapy; OR: odds ratio.

**Table 4:** Comorbidities associated with supplemental oxygen requirement (univariate analysis) in HIV-infected patients with COVID-19 in Argentina (COVIDARE cohort, 2020–2022).

Variable	OR (95% CI)	*p*
Chronic kidney disease	10.95 (4.54–26.39)	<0.001
Chronic steroid use	2.28 (0.25–20.58)	0.46
Heart failure	5.57 (1.31–23.61)	0.02
Other comorbidities	1.75 (1.19–2.59)	0.01

OR: odds ratio.

**Table 5:** Model 2: multivariable analysis of variables associated with supplemental oxygen requirement with discrimination of comorbidities in HIV-infected patients with COVID-19 in Argentina (COVIDARE cohort, 2020–2022).

Variable	OR	95% CI	*p*
Non-tenofovir ART	1.81	0.93–3.54	0.08
History of COVID-19 vaccination (at least one dose)	0.17	0.07–0.43	<0.001
CD4 T-cell count (<350 cells/μL)	4.22	2.34–7.62	<0.001
Age	1.03	1.00–1.06	0.02
Chronic kidney disease	23.61	4.54–122.68	<0.001
Heart failure	0.34	0.02–5.04	0.44
Other comorbidities	1.81	1.03–3.16	0.04

ART: antiretroviral therapy; OR: odds ratio.

## Data Availability

The datasets generated and/or analyzed during the current study are available from the corresponding author on reasonable request.

## Appendix A

**Table A1:** Demographic profile, clinical characteristics, and outcomes of COVID-19 in HIV-infected patients with tenofovir disoproxil fumarate (TDF)- and non-TDF-based baseline therapy in Argentina (COVIDARE cohort, 2020–2022). Values are numbers (percentages) unless otherwise stated.

	TDF (n = 733)	Non-TDF (TAF and Others) (n = 422)	*p*
	n (%) *	n (%) *	
Male sex	479 (65.3)	278 (66)	0.8
Age, years (median; IQR)	44 (36–51)	48 (38–56)	<0.001
Comorbidities	269 (36.8)	173 (41.2)	0.13
Virologic suppression (<20 copies/mL)	583 (79.9)	349 (82.7)	0.24
CD4 T-cell count, cells/μL (median; IQR)	592.5 (430–800)	632 (470–808)	0.06
COVID-19 vaccination (at least one dose)	145 (25.6)	102 (32.6)	0.028
Tomographic abnormalities	97 (13.8)	42 (10.5)	0.11
Hospitalization	115 (16.5)	70 (17.2)	0.75
Oxygen therapy	67 (9.2)	47 (11.2)	0.25
COVID-19 therapy	81 (11.1)	51 (12.2)	0.56
Mortality	7 (1)	6 (1.4)	0.46

* percentages based on cases with available data; TAF: tenofovir alenfenamide; TDF: tenofovir disoproxil fumarate; IQR: interquartile range.
